# 

**Published:** 1901-11-16

**Authors:** 


NOTICE TO CORRESPONDENTS.

All MSS., Letters, Books for Review, and other matters intended for the Editor should be addressed THE EDITOR, "THE HOSPITAL," THE HOSPITAL BUILDING, 28 & 29 SOUTHAMPTON STREET, STRAND, W.C.

The Editor cannot undertake to return rejected MS., even when accompanied by stamped directed envelope.

**Notice to Advertisers and Subscribers.**

All Advertisements, Orders for Copies of THE HOSPITAL, and Business Communications should be addressed to **THE MANAGER (Not to the Editor),** THE HOSPITAL, 28 & 29 Southampton Street, Strand, London, W.C.

The Cost of Subscription, post free, is as follows:—Per week, 2½d.; per quarter, 2s. 8d.; per half-year, 5s. 3d.; per annum, 10s. 6d. Foreign:—Per annum, 15s. 2d., payable, in all cases, in advance.

**NOTICE.—Vol. XXX. of "The Hospital" (April 1901, to September 1901), in handsome cloth binding can now be obtained at the office of this Journal, price, 7s. 6d. Cases for binding the Numbers contained in this Volume 1s. 6d. Index to Vol. XXX., 2½d., post free.**

**The Monthly Part for November is now ready. Price 6d., by post 8d.**

### BOOKS RECEIVED.

T. Fisher Unwin.

"Alcoholism, a Study in Heredity." By G. Archdall Reid, M.B., C.M., F.R.S.E.

Mr. Longhurst.

"Manipulation (or Massage)." By John Andrew Peters.

Marshall Brothers.

"Toward the Sunrising." By J. K. H. Denny.

The Scientific Press.

"Tendencies to Consumption: How to Counteract Them." By J. G. G. Mortimer, M.B.Lond., F.R.C.S.Eng.

Sampson Low, Marston and Co.

"The Nordrach Treatment for Consumptives in this Country." By James Arthur Gibson.

Elliot Stock.

"Scientific Research." By Stephen Smith, M.R.C.S.

### Scraps and Gleanings.

Through the kindness of the friends of the association the 54 girls' branches of the Children's Happy Evening Association will be plentifully supplied with dressed dolls of all descriptions this winter; but the association itself is sadly in need of funds and workers.

During 1900 the number of applications for admission to Dr. Barnardo's Home was 8,795, but of these only 2,879 were actually admitted. Of these 900 were orphans, and 1,430 had only mothers living. The number of children boarded out in country districts for the year amounted to 2,363, and 592 boys and 339 girls were sent out to Canada.

Under the will of the late Mr. T. K. Hardie a sum of over £9,000 has been bequeathed to various hospitals and kindred institutions, whilst £2,000 each has been left to the Refuges for Homeless and Destitute Boys, and the St. Giles's Christian Mission, £1,000 to the Royal Lifeboat Institution, and various other sums to societies for the relief of the poor.

The Countess of Derby has recently opened the Liverpool Sanatorium for Consumption situated at Delamere Forest, Cheshire. This institution is the first of its kind. Between 30 and 40 patients can be accommodated at present in the main building and the adjoining bungalows, and it is hoped that the available accommodation may shortly be increased by the addition of more bungalows, as the administrative part of the building is designed to deal with double the present number of patients. The total cost of the erection and equipment of the sanatorium has been £15,000, which has been provided by Mr. W. P. Hartley, of Aintree, and Lady Willox, wife of Sir John Willox, M.P.

The report presented at the annual meeting of the London Diocesan Police Court Mission, held this year at the Mansion House, stated that in the course of the past year 21,103 visits had been paid to or on behalf of offenders; 1,613 total abstinence pledges had been taken; upwards of 2,700 persons had been placed out in the world, and 6,755 had been materially helped. The society has purchased a plot of ground at Yiewsley for the purpose of erecting a home for boys from 17 to 20 years of age charged at the courts. A sum of £2,160 is still required for this home, and an earnest appeal is made to enable this all-important branch of the work to be carried through. A sale of work in aid of the Mission will be held at the Portman Rooms on December 3rd.

### The Student of Medicine.

### APPOINTMENTS.

**Edmund L. Anderson**, M.B., Ch.B.Vict., L.S.A.Lond., appointed Honorary Assistant Surgeon to the Hospital for Cancer and Skin Diseases, Liverpool. **G. A. H. Barton**, M.R.C.S., L.R.C.P., appointed Honorary Anæsthetic to the City Orthopædic Hospital. **J. H. Battersby**, M.D., C.M.Edin., appointed Honorary Surgeon to the Doncaster Infirmary and Dispensary. **Elmore Brewerton**, F.R.C.S.Eng., appointed Assistant Surgeon to the Royal Westminster Ophthalmic Hospital. **Arthur G. Brinton**, M.R.C.S., L.R.C.P., appointed Resident Surgical Officer to the Birmingham and Midland Eye Hospital. **J. Walter Brown**, M.B., B.Ch.R.U.I., appointed House Surgeon to the Royal Victoria Hospital, Bournemouth. **J. Basil Hall**, M.C.Cantab., appointed Honorary Surgeon to the Bradford Royal Infirmary. **John A. Hogg**, L.R.C.P., M.R.C.S., appointed Medical Officer of Health to Castle Donington Rural District Council. **E. L. Hunt**, L.R.C.S., L.R.C.P.Irel., appointed Certifying Factory Surgeon for the Sherston District of Wilts. **T. H. P. Morris**, M.R.C.S., L.R.C.P.Lond., appointed Certifying Factory Surgeon for the Halesworth District of Suffolk. **Christine M. Murrell**, M.B., B.S.Lond., appointed Assistant House Physician to the Royal Free Hospital, London. **W. P. O'Meara**, L.R.C.P., L.R.C.S.Edin., appointed Medical Officer for the No. 1 District of the Southampton Union. **Marion J. Ross**, M.D.Glasg., appointed House Surgeon to the Morpeth Dispensary. **Walter Sykes**, L.R.C.P.andS.Edin., L.F.P.S.Glasg., appointed Junior House Surgeon to the Birmingham and Midland Eye Hospital. **Alexander Trotter**, M.B., Ch.B.Edin., appointed Senior House Surgeon to the Birmingham and Midland Eye Hospital. **C. E. Whitington**, M.R.C.S.Eng., L.S.A., appointed Certifying Factory Surgeon for the Tuxford District of Notts. **Florence Elizabeth Willey**, M.B., B.Sc.Lond., appointed Assistant House Surgeon to the Royal Free Hospital, Gray's Inn Road. **James Mitchell Wilson**, M.D., C.M.Glasg., appointed Medical Officer of Health to the East Riding County Council.

